# Editorial

**Published:** 1900-04-15

**Authors:** 


### EDITORIAL.

We have been asked to reply editorially to the following question: "Is a member of the regular dental profession, who is in good standing in the societies, justified in testifying in case of a mal-practice suit in behalf of a rank advertiser?" The manner of statement of the question is so faulty that it is with difficulty that we comprehend the intention. If it means to inquire whether a reputable dental practitioner should endeavor to screen a rank advertiser from the consequences of his ignorance or carelessness, even though he be practicing regardless of all ethical considerations, we should give the same answer as in the case of the most scrupulously ethical practitioner. The object of a legal trial is to establish the facts in a controversy, and witnesses are expected to assist the officers of the court in every way to arrive at definite and equitable conclusions. We hold, therefore, that prejudice of any kind should have no influence in modifying the statement of facts involved. We should rise to the high privilege of helping to establish truth whenever called upon to do so, whether this may be at the bar of justice or on the public platform. Tell the truth, the whole truth and nothing but the truth.

The suit to determine the validity of the patent on the "Donaldson Brooch," has resulted in a decision against the patent. As to the equities in this matter we are uninformed. But it does seem that the price asked for these brooches is out of proportion to the expense of manufacture. And it was perhaps a mistaken policy on the part of the manufacturer to put the price so high that immitation of an inferior kind have been placed on the market. A reasonable compensation in this, as all other cases, would have been more profitable to the patentee and consumer in the long run.

### DIPLOMACY AND DENTISTRY.

"We learn through an Associated Press report, which has been extensively printed in the daily press of this country, that certain individuals with more energy and judgment, have, through political influence, secured an audience with the Secretary of State, and obtained his promise to instruct our ambassador at the court of St. James, to endeavor to remove the restrictions imposed by the dentists' act of England, which operates against the admission of holders of the American degree, to practice in Great Britain.

Whatever may have been the inspiring motive of the act, it can only be regarded, by any one at all familiar with the equities of the case, an impertinence which will be justly resented by the dental profession of England, and deplored by all American dentists who have a proper respect for the basal principles of dental legislation. We can not for a moment believe that our ambassador will permit himself to be placed in a position that is illogical in view of the principles at stake, and ridiculous upon its face. We could certainly resent any diplomatic interference with our educational standards by the representations of another nation. And yet these superservicable parties without a visible constituency to support their action, have taken it upon themselves to do a thing which would be discreditable to American dental interests were it not apparently, and as we hope it is wholly, an individual expression of mental vagary."

Editorial in *Dental Cosmos* for April. We have not seen the press reports which furnish the basis for the above editorial, neither have we the personal knowledge of the matter that would enable us to shed any new light on it. Brother Kirk's characterization seems rather severe, and yet the provocation, in view of what is being done to come to some understanding on educational matters with foreign countries on another basis of effort, may justify the language used.

Within the past year a movement was inaugurated looking to the consummation of a better understanding as to the qualifications of the American dentist in Europe, and events of a perfectly normal character seem now to be transpiring, which will ultimately accomplish all that is reasonable in this direction. We are coming to a better understanding in our own country and among ourselves as to what shall constitute an educated profession. There never was a time when there was so intense earnestness on the part of dental educators to provide facilities in the way of buildings, trained instructors and other helpful resources for imparting knowledge and developing technical skill as at present. And this development is not disregarded in the older countries. And it may be just as well for us to wait until we have become full-grown before we attempt to force our attentions on those who have realized our deficiencies perhaps as we do not. We are firmly of the opinion that this matter will finally be adjusted on an educational basis. And that the sooner American educational institutions come to an agreement and establish an adequate standard for a professional education and teach up to it, the sooner our schools and graduates will be recognized not only at home but elsewhere. Let us have a uniform curriculum and a uniform standard. And then we will be in a position to go to other countries and demand in the name of our standard such recognition as we are entitled to.

---

Mr. Morton Smale, dean of the London Hospital Dental School and Mr. E. Lloyd Williams, professor of mechanical dentistry in the same school, have made recently a visit to the principal schools of this country. They are to have a new building, and no doubt will profit by witnessing the completeness of some of our recently erected and equipped educational plants. We found them very pleasant and agreeable gentlemen, earnest and full of devotion to the profession, and especially its educational aspect.

